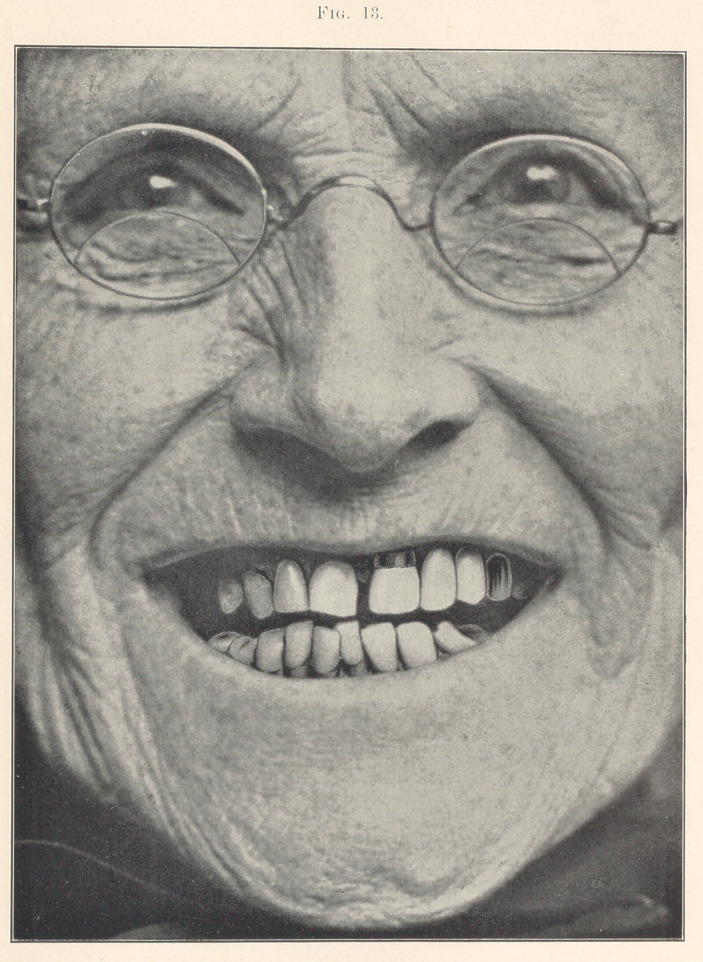# Facial and Dental Harmony

**Published:** 1902-02

**Authors:** Lawrence W. Baker

**Affiliations:** Boston, Mass.


### International Dental Journal.

Vol. XXIII. February, 1902. No. 2.

### Original Communications.[1]

### FACIAL AND DENTAL HARMONY.[2]

BY LAWRENCE W. BAKER, D.M.D., BOSTON, MASS.[3]

Mr. President and Fellow-Members of the Harvard Odontological Society,—I know that it is considered poor form to begin a paper with an apology, but I am sorry to say that is my condition this evening, as what I am to give you, gentlemen, is nothing more nor less than I give the students at the school. I might also add that these charts I had prepared for school use, and it is through the kindness of Dean Smith that we have them here to-night. With your permission I will read much of what I have to offer, as I think that I can give it in a much more connected manner than by speaking from my notes.

While a student at school and since I have been in private practice I have made somewhat of a study of artistic artificial work. I will endeavor to give you to-night the results of my investigation. I have purposely avoided giving anything that you can find in

[1] The editor and publishers are not responsible for the views of authors of papers published in this department, nor for any claim to novelty, or otherwise, that may be made by them. No papers will be received for this department that have appeared in any other journal published in the country.

[2] Read before the Harvard Odontological Society, April 25, 1901.

[3] Instructor in Orthodontia and Lecturer on Artistic Artificial Work at the Dental School of Harvard University.

text-books, and will endeavor to confine myself strictly to the subject.

Possibly you gentlemen might be interested to hear how I worked out some valuable points for myself regarding the work. While studying at school I had occasion to make an entire upper gold denture for a middle-aged woman. As you know, students are not always known to do the correct thing, so I made a mistake by getting the teeth too long, so much so that I was afraid that I would have to do the work over again; but I was instructed to try and shorten the teeth by grinding, so I went to work with a coarse stone and the engine. I was very much worried for fear I could not restore the even, graceful curves of the cutting edges of the teeth.

After taking off about one line I tried them in, and to my surprise they looked a great deal better than they did previous to grinding; in fact, they looked so well that I was afraid to even touch them with the stone. Now, a question that naturally came to my mind was, What had I done to cause such a marked change? You must remember that I had not attempted to get the cutting edges even, but simply to hack them off, so to speak, leaving them rough and uneven. It was that I had done in a short time what Nature is years in accomplishing,—that is, wearing the teeth. It simply made the teeth harmonize with the age of the patient more than before.

After noticing this I took pains to examine natural teeth as well as those of casts, and to my surprise I found that in most cases of patients having reached the age of twenty years there was a wearing of the teeth more or less according to the age of the person. I would like every one of you to notice and see if this is not so. You might observe the different ways that I have ground these and the various effects that have been produced by so doing.

Figs. 1, 2, and 3 (see illustrations) are identical in form, being taken from the stock of S. S. White, mould 62. I do not think that any one of you would say that they were originally of the same form, being so different in character. You see that by grinding each tooth a little different from its fellow that awful sameness is broken up; that is, each tooth is given a certain amount of character and individuality.

We will now turn to the charts, and I will endeavor to explain what I have been reading about. As I was just reading, Figs. 1,

Fig. 1.

Fig. 2.

Fig. 3.

Fig. 4.

Fig. 5.

2, and 3 were made from the identical mould, and I venture to say that I do not think that an ordinary observer would mistrust that they were the same teeth, so far as the form goes, as they differ so in character.

Fig. 1 is a graceful case. Notice that all the curves are even and in harmony with each other. There is no irregularity in the alignment of the teeth to break up the artificiality. I have tried to do it merely by grinding each tooth a little different from its fellow, adding individuality. Notice that the cutting edge of one central is slightly concaved, while the general shape of the cutting edge of its mate is convex. Observe also that these teeth vary slightly in their approximal surfaces. I have ground the edges of the laterals and cuspids as well. You notice a great deal can be accomplished towards breaking up the artificiality by making these slight variations; that is, the hard, set appearance is destroyed.

Fig. 2, you will observe, is not so graceful as Fig. 1, and that the teeth are ground in a little different manner. There is a squareness given to the approximal surfaces of the centrals, also the cutting edges of the teeth are ground in shorter curves.

We will now take up Fig. 3, which I consider is fully as natural as the two preceding ones. Notice that the symmetry is entirely wanting; take for example the centrals. See that they differ in length, also the slightly uneven grinding on the cutting edges. The difference between the laterals is quite marked. Notice that the right one is worn much more than its mate, which is worn scarcely any, as it does not come in occlusion with the lower teeth. See the variation in the cuspids. Notice how the right one is represented as having much more work to do than its fellow, as shown by its extensive wearing, which is represented better in the next chart. Also notice how the strike has forced it up, and that its general direction differs from the left. Of course, in a case like this we will assume that the lower teeth are somewhat irregular to produce these results.

Fig. 4 represents the three cases that I have just been talking about in a relative way. The first or top case is one, the middle, the third, and the lower one, case two. We will take the cuspids first. In the upper set there has been scarcely any grinding at all, while in the middle one there is considerable, the distal half of the cutting edge being ground to represent a regular facet made by the occlusion with the lower teeth, while in the lower case we have ground about as much as the first, although in a little different manner. Also notice the alterations in the laterals.

While I am on the subject of the cutting edge it might be well for me to explain the rest of the cases from this stand-point.

We now come to Fig. 5, which represents an upper and lower denture for a man of about thirty-five years of age, in occlusion. He is supposed to be a large, strong, wiry sort of fellow, with powerful angular jaws. I have tried to make the teeth harmonize with this type of face. We will see how I have treated the cutting edges to carry out these lines. There is a general idea of squareness given to the cutting edges which brings out the angularity. See that the cutting edges are about on the same plane, also that the mesial angles of the centrals and especially the laterals are quite sharp, which enhances the angularity. Possibly by comparing this and the next chart, Fig. 6, these ideas may be shown by contrast, as this is for a mild, easy-going sort of person. See how the mesial corners of the laterals are rounded, which gives mildness to the case. Passing to the lower set of Fig. 5, notice that there is the same straightness across the cutting edges, which is in keeping with the principle of angularity. Notice that the edges are not ground square across, but chiselled from before backward, as they would be in nature from their sliding against the lingual surfaces of the upper teeth. In this illustration the mouth is represented as being slightly opened, so that the cutting edges of the lower teeth can be seen.

Fig. 6, as I have already said, is for an easy-going sort of fellow, but a somewhat older person than figures in the preceding case, say about fifty years of age. Notice that the general tone of the case shows mildness and deliberation, while the preceding case shows strength and angularity. I think that the principal difference is represented in the way that I have treated the cutting edges. I have also attempted to demonstrate another feature in this case, and that is the action of acid saliva on the cutting edges combined with wear. We all know that if the dentine is exposed to slightly acid saliva it will be attacked quicker than the enamel. The result is that when the dentine between the two plates of enamel is exposed by wear, it would be eaten away, so to speak, leaving the two walls of enamel unsupported, which by constant use will become chipped, giving a ragged, eaten appearance to the cutting edges, and that is what I have tried to show in this case, especially so in the centrals.

Fig. 6.

Fig. 7.

Fig. 8.

Fig. 9.

Fig. 10.

Fig. 11.

Fig. 7 does not present any feature that I have not already tried to explain unless it is what we might call the "thread tooth." In spite of what we can do some of our women patients will bite threads, the result being that the enamel of one or more teeth will become nicked. I have observed that the left lateral and cuspid suffer most often, as shown in Fig. 8 by the slight nicking of these teeth. There is also the same wearing on the cutting edges that, no doubt, by this time is beginning to wear on you, as I have said so much about it.

Fig. 9 is supposed to represent a denture for an elderly gentleman of about sixty years of age, which, as you will expect, shows the effects of the occlusion perhaps more than any other case that I have here. Not only do the cutting edges show the wearing, but the cusps of the bicuspids and molars have almost entirely disappeared.

Figs. 12 and 13 are photographs taken directly from nature. They appeared in a series of faces shown in the January issue of the *Dental Cosmos.* As these are absolutely true to nature I do not consider that I have been wasting time talking about grinding the cutting edges.

Fig. 12 represents the face of a man of about fifty-nine years of age. The lower teeth are abraded nearly one-half their length. The dentine, which is exposed, has become much stained by the use of tobacco.

Fig. 13 represents the face of a woman of about seventy-one years of age. The upper and lower teeth are natural excepting the upper left central, lateral, cuspid, and bicuspid, which I should consider was a piece of bridge-work. These artificial teeth are about as they came from the stock, so far as the cutting edges go; that is, they have not been ground to show the wearing. I will cover all the natural teeth, leaving the artificial ones exposed. Now I will cover the artificial ones, leaving the natural teeth exposed to view. I think that you will all be surprised with the effect,—that is, how much relation there is between the facial and dental harmony. I do not want you, gentlemen, to think that I am criticising this bridge-work, because, no doubt, at the time that it was made the natural teeth were worn scarcely at all, and I have no doubt that at that time there was better harmony between the artificial and the natural teeth.

In grinding that same set that I have just mentioned,—that is, the one that I made when a student,—I did something else to improve the appearance. The teeth were too light in color at the tips. Now, by grinding this off, it resulted in improving the color, making it harmonize also with the age of the patient. One great trouble with dentists is that they get the teeth too white to fit elderly patients, and it is just this class of people that we have to supply dentures for most often. I would like to impress the following points on every one of you here this evening: *That the teeth age with the patient both in wearing and in color, and it is by imitating nature in these two respects that the key-note of success, artistically, in artificial work is reached.*

As a person advances in life it is shown in various ways. We all know that the hair either comes out or turns gray. The skin becomes wrinkled. There is even a change in the color of the skin. Now, why do not the teeth show this aging? That is just what they do. To place white teeth in the mouth of an elderly woman would be the same as putting a wig on her made for a woman of twenty years of age. You can imagine how it would look,—out of place and false; just the same as the teeth for a woman of twenty would look in her mouth. The reason why they would look false is because there is a lack of harmony between the age of the patient and the apparent age of the teeth.

We now come to another important consideration,—namely, the festooning of the gum line, which plays a very important part in the general effect of the case. This must, of course, harmonize with the rest of the work. If the lines of the teeth are even and graceful, so must the gum line be. If it is for a rugged, strong man, the gum line must follow being rugged and strong, or the character of the case is lost. For a lazy, sluggish, lymphatic person the gum line wants to be low and flat.

Here is another place where the sameness of the teeth can be broken up as by grinding,—that is, in varying the festoon. We can also age the teeth by making them somewhat, what we call, "necky," by making a recession of the gum that we so often see with advancing age. It is for this reason that the manufacturers have some moulds made with long necks.

We will now turn to the charts again, and I will endeavor to explain what I have been reading about the gum line. Take Fig. 1, which shows a well-balanced case. Notice that the festoons are even, graceful curves, and in harmony with the general lines of

Fig. 12.

Fig. 13.

the teeth. In Fig. 2 we see that the gum line is less graceful. Fig. 3 shows how we can break up the monotony in the appearance of our work by varying the gum line as we did the cutting edges. See that the left central is somewhat shorter than its mate; also that the gum line is a broken curve. There is a difference in the festoons over the cuspids. No doubt, now that I have mentioned these variations, you may think that I have been exaggerating them in this case, but taking the case as a whole I do not consider that I have.

Notice how in Fig. 5 I have tried to bring out the strength and angularity of the case by the gum line, since it is for a strong, angular, square-jawed man. See that, instead of the line having general curves, it is made up of short straight lines, which are well shown over the upper anterior teeth. Notice also that the length of the cuspids is increased by the height of the festoons, which adds strength.

In the lower set much character is given the otherwise plain case by the gum line alone. Over, or rather under, the centrals the line is lower than at the laterals. Then, when we come to the cuspids there is a decided drop. Now, by covering the gum line with the pointer we will notice how it weakens the case, and by uncovering the gums the strength returns. At the same time these slight variations help to break up the monotony of the case.

There are no new features in the next case, shown in Fig. 6, but notice that there is harmony between the cutting edges of the teeth and the gum line; that is, they both go to produce mildness.

In Fig. 7 I have shown a recession of the gum over the left central which we will suppose was caused by vigorous brushing of the teeth. No doubt you all have observed that in most cases where the recession is caused by too much brushing, it is the left side that suffers most, as with a right-handed person it is the easiest side to get at. Also observe that as the teeth are quite long, we have the gum line in keeping with this idea; that the arches of the festoons are high, which point might be brought out more clearly by comparing with the preceding case. In Fig. 9, for the elderly gentleman, the gums are very much receded, giving the appearance of looseness to the teeth.

We now come to the gum itself. For a patient that does not show the gum in talking or laughing it is of little importance; but in those cases where the gum is shown, and it does not harmonize with the surrounding, the artificiality is at once discovered. In a case of this description the way to do would be to work the gum up carefully in wax and have block teeth carved by hand. In this way the correct arrangement of the teeth can be obtained as well as a natural-looking gum.

Possibly you have noticed the prominences over the roots, especially over the cuspids, showing the cuspid eminence, which gives so much character to the mouth and even to the corner of the nose. Now, if the plate line does not run high and the prominences are not sufficient, there will be a drooping at the angle of the nose. Then, again, a great deal of strength can be shown by making the prominences over the roots pronounced. For a strong and robust person the roots are strong and well developed, as shown in Fig. 5.

In the illustrations you will notice that the highest and fullest point of the gum is over the cuspid, to build out the part of the face lost by the extraction of that tooth, which is the most important tooth, from the artistic stand-point at least, on account of the excessive length of its root, which is clearly shown in the photograph of the section of the skull (Fig. 11). We can easily see that when this tooth is extracted not only will the expression of the mouth be effected, but that of the nose as well, as the muscles of expression in this region will be distorted, giving a very unnatural look to the face. This point my father, Dr. H. A. Baker, has called to my attention many times.

I would like to say a little about the temperaments, but do not want to take up much of your time, as this can be found in detail in the text-books. I will give enough so that you can better appreciate the cases that I have here this evening, for no one can be successful in this work unless he thoroughly understands the divisions of temperament and can recognize them when he sees them.

I have only one or two cases that would be called pure types. The nervous is the most typical, which is shown in Figs. 7 and 8; the sanguineous, Fig. 1, is also quite good. The salient points regarding the temperaments are that we are classified according to our characteristics into the sanguineous, bilious, nervous, and lymphatic types and their various combinations.

The characteristic features of the teeth of the sanguineous temperament are that they are well proportioned, abounding in curves, with rounded outlines and well-proportioned cusps. The color is of a creamy yellow inclined to translucency. In the bilious temperament the teeth are large and inclined to angularity, rather long in proportion to the breadth, the color being a pronounced bronze yellow. In the nervous type, length predominates over breadth, abounding in very graceful curves with fine, long cutting edges and cusps, the color being pearl-blue or gray inclined to transparency. The lymphatic temperament is the complete opposite of this, as the teeth are large, unshapely, very broad cusps and poorly defined, the color being pallid, opaque, or muddy.

We have thus far studied the cases in parts; we will put these together and consider the effect of the cases as a whole. I trust that you will excuse me if I repeat a great deal of what I have already said.

CASE 1. For a man of about thirty years of age having somewhat of a sanguineous temperament. The general impression of the case is graceful. Note there is some wear on the cutting edges of the anterior teeth, especially the centrals; one is slightly concave, while the other is convex, as I have already explained on the chart, breaking up that sameness that is so liable to enter into this work. See that the gum margin is graceful and in harmony with the rest of the case and that the color is of a creamy yellow. This case as well as the two following are made from mould 62, S. S. White.

CASE 2. Made from the same mould as Case 1, but notice that the character is entirely different, showing that it is not the manufacturers that are wholly to blame for the sameness of our work. Notice the effect produced by grinding the cutting edges,—that they differ entirely from Case 1. Also observe the slight elongation of the centrals, the shape of the gum line, and contrast it with the preceding case. This is for a man of about thirty to thirty-five years of age, of a somewhat sanguo-bilious temperament, the color being dark yellow.

Case 3 I really do not know just how to classify, but I should say that the bilio-lymphatic temperament predominated. The case is supposed to be for a man with strong jaws, but of a different type from Case 4, as that was for angularity, while this is for a more corpulent person.

CASE 4 (shown in Fig. 5). For a man of about thirty-five years of age of a bilious temperament, having a strong, angular build. Observe how the general effects of the case carry out that idea. There is just enough grinding on the cutting edges of the teeth for the age of the patient considering the density and hardness of the teeth. I might stop to say that a great deal of wearing depends upon the quality of the teeth and the chemical reaction of the saliva, as will be shown in the next case. See that the artificiality is broken up by the slight lapping of the superior laterals over the centrals, and that the cutting edges are about on the same plane. Observe that the gum line differs from the preceding cases, it being more angular. In the lower there is a slight lapping of the anterior teeth, also the cutting edges are worn not flat, but chiselled from the fore backward as we find them in nature. Notice that the festoons of the lower cuspids are much lower than the other festoons, giving character to the otherwise plain case. The mould is 181, S. S. White.

CASE 5 (Fig. 6). For a man of about fifty years of age of a sanguo-lymphatic temperament. The character of this case is in the grinding of the cutting edges of the anterior teeth to show the effects of acid saliva combined with wear. Observe that they are ground to give the appearance of their being somewhat uneven and ragged. You see that the prominences over the roots are very slightly developed. There is a slight space between the centrals; the mesial corners of the laterals are rounded, which is not especially graceful, but often met with in nature, especially for a person of this type, adding mildness to the case. The color is of a muddy yellow. The mould is 35, S. S. White.

CASE 6 (Figs. 7 and 8). This is a typical nervous type for a woman about forty years of age. She is supposed to be one of those highly organized, angular-featured woman, one whose face is narrow and sharp. Now notice how characteristic the denture is for a person of this description. See that the arch is quite pointed, with a general pitching forward and crowding of the teeth. Notice that the festooning is in harmony with the idea of the case. Additional character is given by replacing the smaller centrals of the set by much larger ones. Observe that the fillings are where they would occur in nature. The left first bicuspid is supposed to be a banded crown. The color of the teeth is a bluish gray. By comparing the difference between this and the preceding case, I think that you will see the amount of character that can be given by the teeth, and that what I have said about the harmony of the facial and dental lines is true. This case is made from mould 100, S. S. White.

Case 7 (shown in Fig. 9) is a lower denture for an elderly gentleman who has been used to high living. See that the teeth are somewhat irregular, but not enough so to attract attention. Notice how the age of the case is shown by the extensive grinding on the cutting edges. See the length of the teeth and how the gums have shrunk away from the necks. Also observe that the gums are somewhat puffy and give the appearance of being inflamed and gorged with blood.

I consider that I was complimented the other day on this case, for when a friend, who is a dentist, was in the office and examined it, he remarked, "You had better hurry up and give this paper before the old fellow dies of age." This pleased me, because it was the idea of age that I was striving for in this case. The mould is 40, S. S. White.

There is one more point that almost slipped my mind, and that is about the use of stains. Dr. A. H. Parker, who does some very artistic work in this direction, gave me my first ideas on this subject.

One of the troubles with stock teeth for partial work is that it is almost impossible to get the color. The general color may be good, but to get some of the gradations that we find in nature is, I think, almost impossible. If one, however, is expert with the use of stains, his work will be much more satisfactory in appearance. We can imitate the teeth that have a mottled color. Then we sometimes find them with spots of a lighter shade than the general color. I think, however, that the most pleasing work in staining is to reproduce the tobacco stains that we find on the teeth of inveterate smokers. It is almost impossible to get a natural effect in partial work in one of these mouths unless we resort to the staining, for the artificial teeth will be so conspicuous that the eye will detect them at once. Now, by copying the tobacco stains on the remaining natural teeth the artificiality will escape unnoticed. I am, of course, referring only to those cases where the teeth are exposed to view. I myself do not consider that it is really necessary to be so particular as to staining the posterior teeth unless they show under certain conditions. The photograph (Fig. 10) that I have here gives but a very poor idea of the staining, which shows much better in the specimens that are being passed around.

I believe that this is about all that I have to offer this evening, gentlemen. I feel that I have only just begun this work, and there is no doubt but that after a few more years of study I will be ashamed of what I have said to-night.

I trust that you appreciate the fact that I have presented only seven or eight different cases, and that it would be impossible for me to do justice to so large a subject with such a small amount of material.

I feel sure that during the discussion there will be many points brought up that I have not touched upon at all. I wish to thank you, gentlemen, for your kind attention to me this evening.

## Figures and Tables

**Fig. 1. f1:**
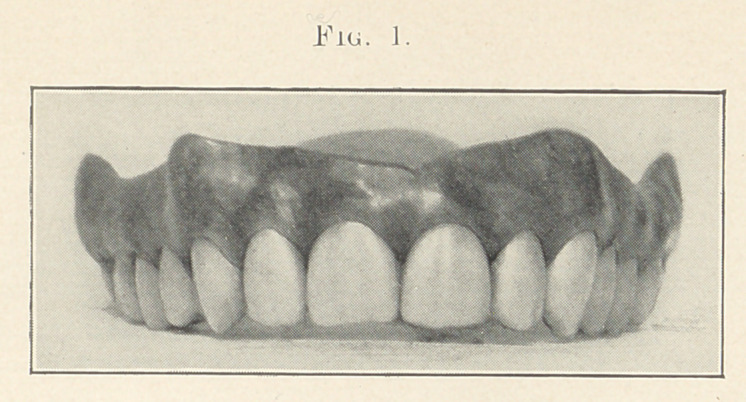


**Fig. 2. f2:**
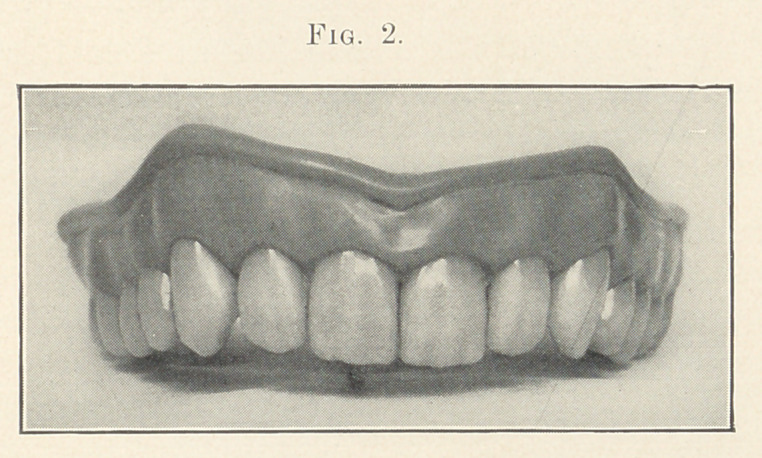


**Fig. 3. f3:**
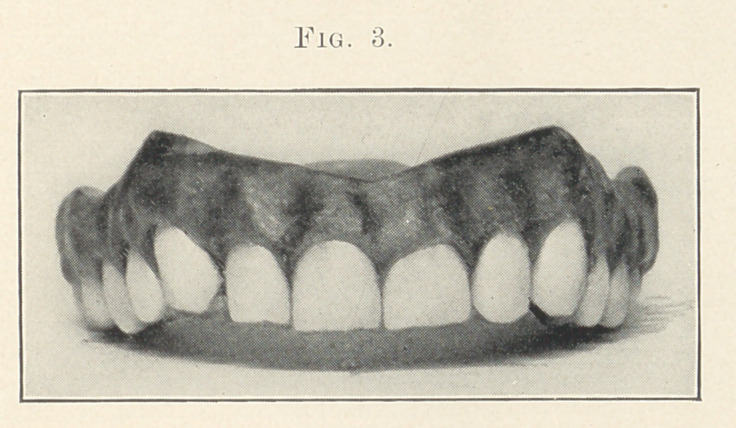


**Figure f4:**
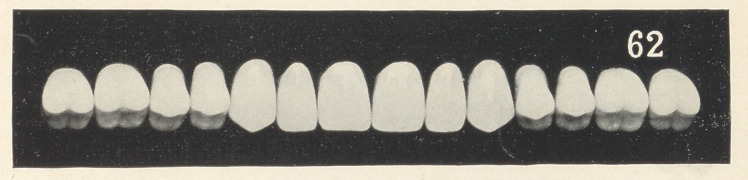


**Fig. 4. f5:**
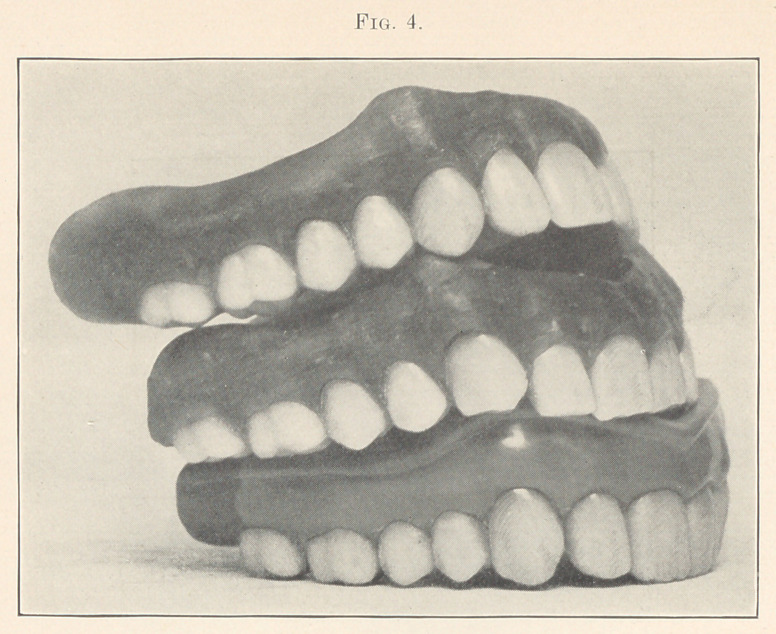


**Fig. 5. f6:**
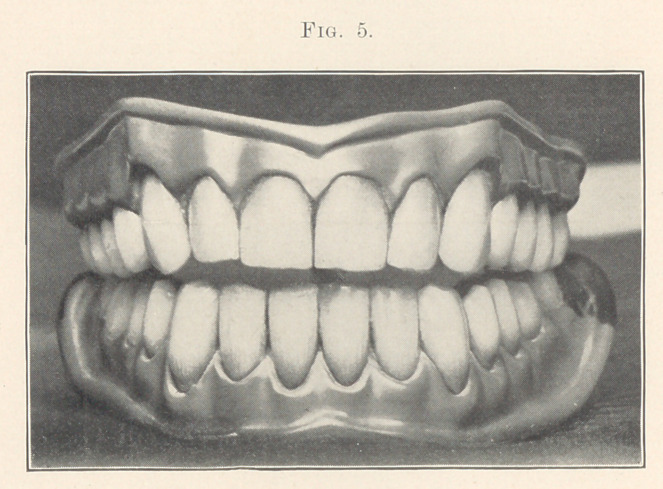


**Figure f7:**
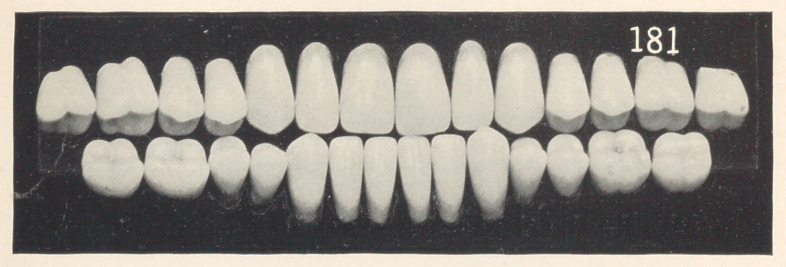


**Fig. 6. f8:**
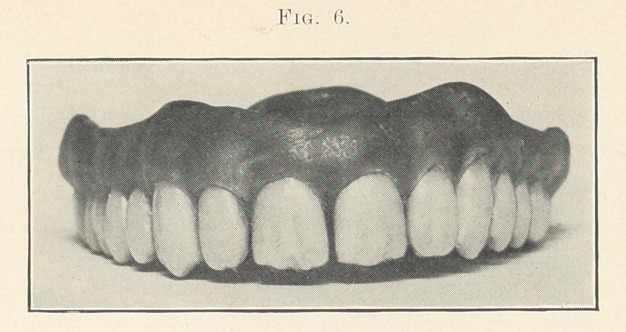


**Figure f9:**
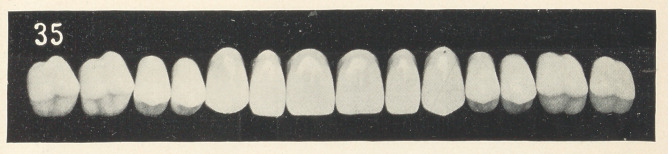


**Fig. 7. f10:**
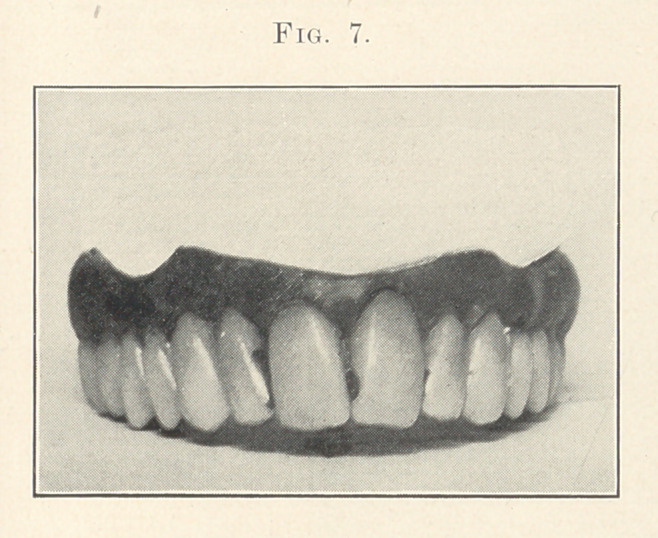


**Fig. 8. f11:**
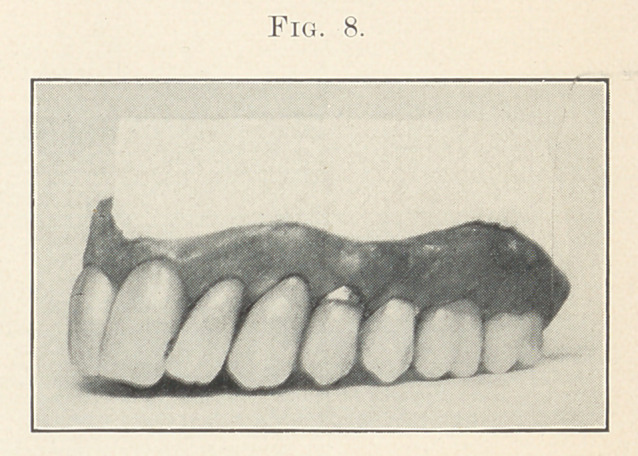


**Figure f12:**
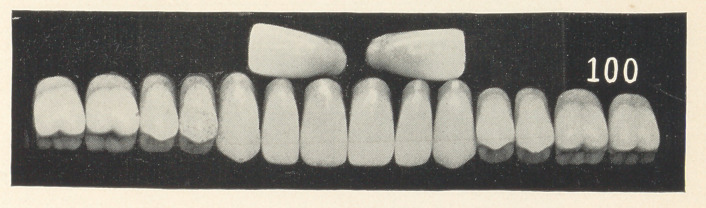


**Figure f13:**
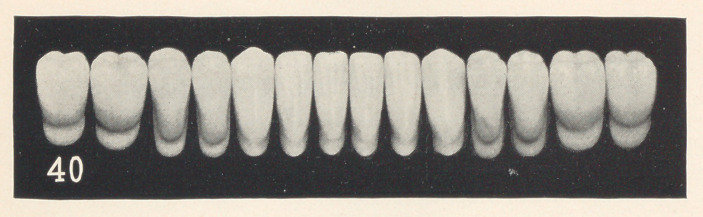


**Fig. 9. f14:**
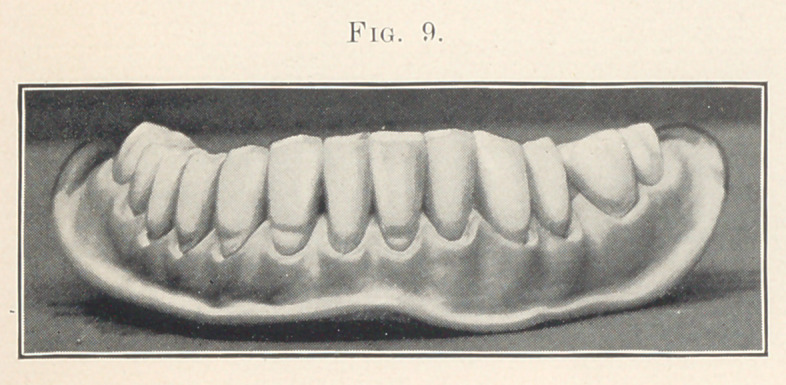


**Fig. 10. f15:**
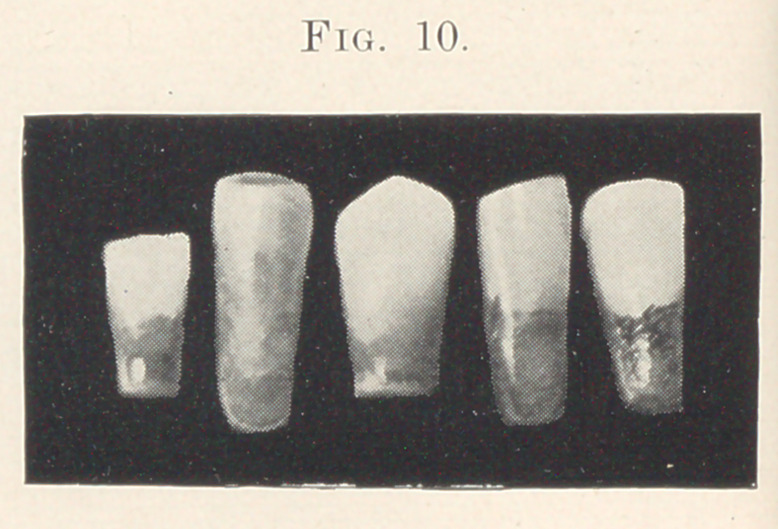


**Fig. 11. f16:**
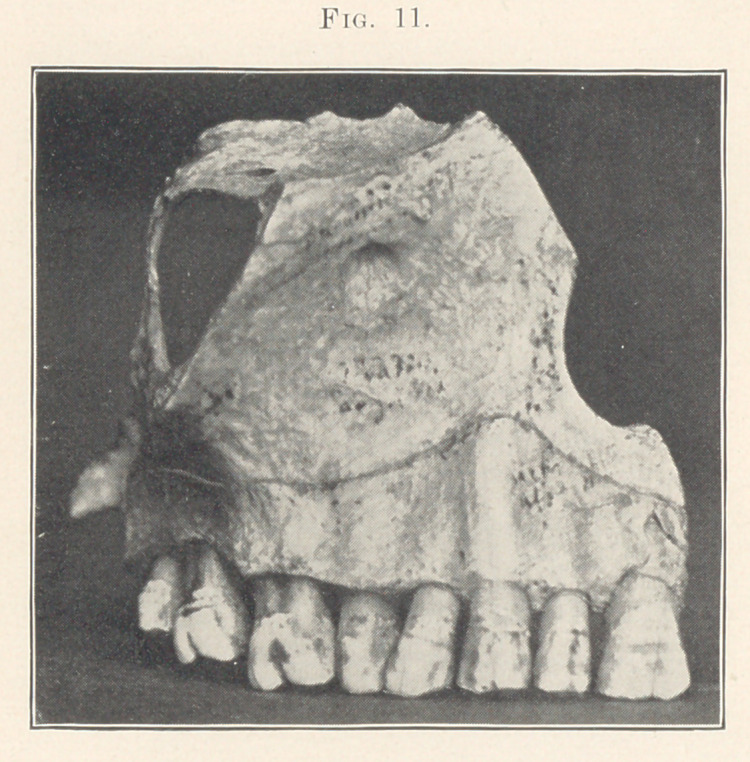


**Fig. 12. f17:**
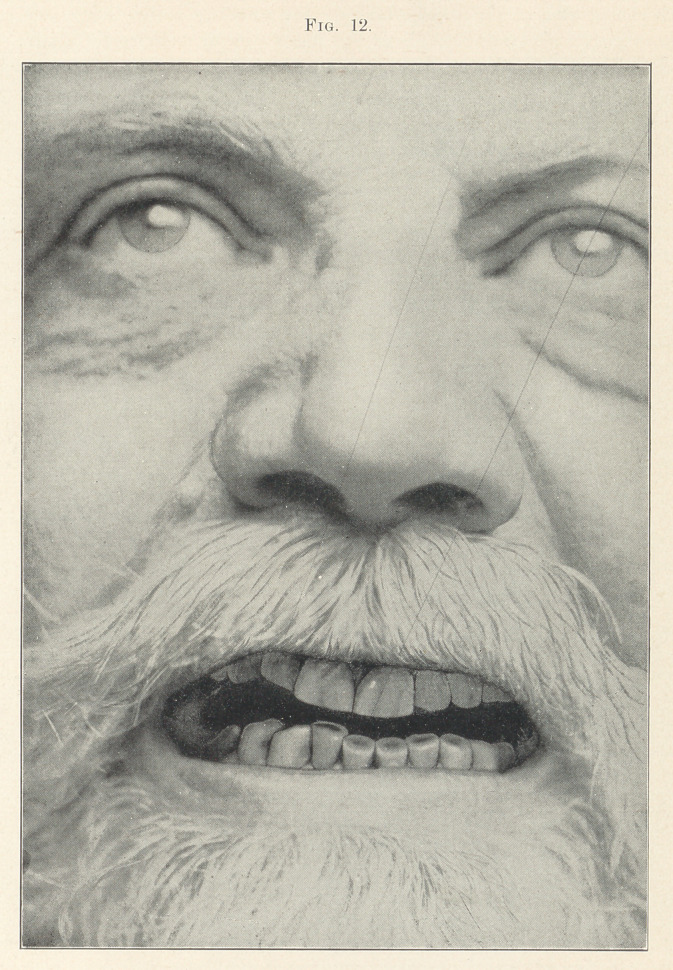


**Fig. 13. f18:**